# Progressive increase in cavitation with the evolution of fungus ball: A clue to the diagnosis of chronic necrotizing pulmonary aspergillosis

**DOI:** 10.4103/0970-2113.53235

**Published:** 2009

**Authors:** R. Prasad, Sanjay R. Garg

**Affiliations:** *Department of Pulmonary Medicine, King George's Medical University, Lucknow - 226 003, UP, India*

**Keywords:** *Aspergillus*, diabetes, tuberculosis

## Abstract

Chronic necrotizing pulmonary aspergillosis (CNPA) is an uncommon pulmonary infection seen in the patients with chronic obstructive pulmonary disease, bronchiectasis, pneumoconiosis, diabetes mellitus, alcoholism, poor nutrition or low dose corticosteroid therapy. Here, we are presenting a case of CNPA with diabetes mellitus that was misdiagnosed as pulmonary tuberculosis.

## INTRODUCTION

A variety of clinical entities caused by the fungus aspergillus have been described. The spectrum Aspergillus lung disease includes saprophytic aspergillosis in the form of pulmonary aspergilloma, immune disease in the form of allergic bronchopulmonary aspergillosis and hypersensitivity pneumonitis, and infectious disease in the form of invasive and chronic necrotizing or semi-invasive pulmonary aspergillosis. In this report we present a case of diabetes mellitus with progressive increase in cavitations on chest radiograph that was diagnosed as a case of chronic necrotizing pulmonary aspergillosis.

## CASE REPORT

**Figure 1:**
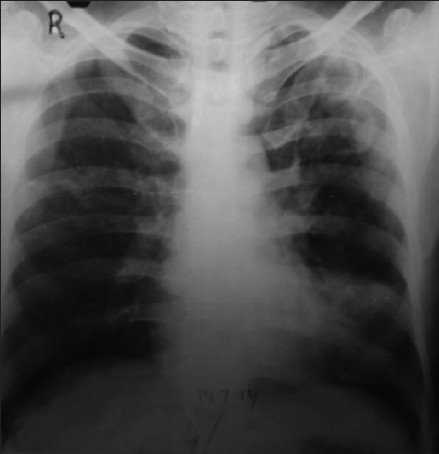
Chest radiograph showing left upper lobe cavitary infiltrate

A 40 year old male who was known to have type I (insulin-dependent) diabetes mellitus, presented with complaints of productive cough, breathlessness, fever, weight loss, and hemoptysis for three years. His previous chest radiograph revealed left upper lobe cavitary infiltrate [Figure 1]. His routine investigation revealed uncontrolled blood sugar and negative sputum smear for acid-fast bacilli at that time. Patient had received two years of antituberculosis treatment along with insulin from a private practitioner, though his sputum did not show any acid-fast bacilli. Despite adequate antitubercular treatment, he deteriorated clinically as well radiologically.

**Figure 2:**
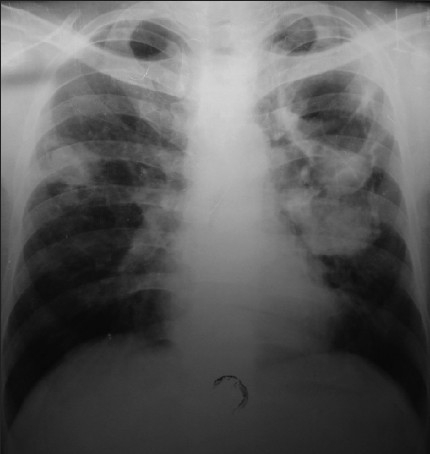
Chest radiograph after two year of antitubercular treatment showing increased cavitation along with fungus ball and progression of disease to right side

**Figure 3:**
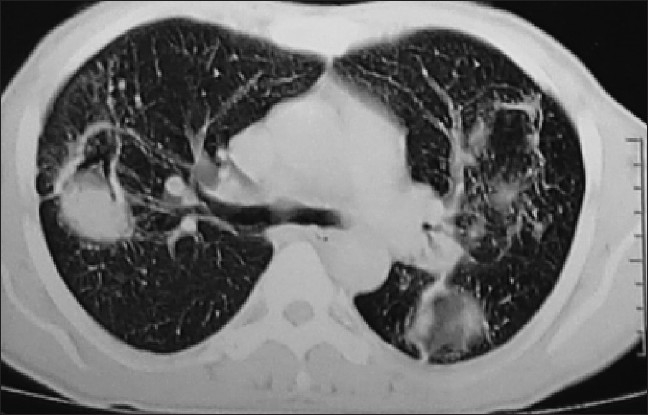
CT scan of the chest showing a crescent shaped lucency (air crescent sign) with in the area of consolidation right middle lobe

**Figure 4:**
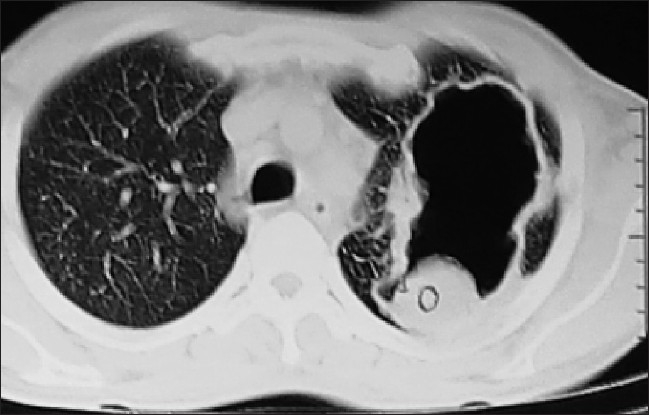
CT scan of the chest showing a crescent shaped lucency (air crescent sign) with in the area of consolidation left upper lobe

On admission, general examination revealed grade III clubbing of fingers and toes. Examination of respiratory system revealed bronchial breath sound over left mammary area and coarse crepts in bilateral suprascapular region. Review of his serial chest radiograph revealed progressive increase in cavitation with the evolution of fungus ball and progression of disease on right side [Figure 2]. His hematological and biochemical investigations were with in normal limits except uncontrolled blood sugar (fasting blood sugar: 202mg/dl and post prandial: 368mg/dL). Enzyme-linked immunosorbent assay (ELISA) for human immunodeficiency virus was negative. Multiple sputum smears revealed no bacteria and acid-fast bacilli. The culture by BACTEC did not show any mycobacteria. The sputum on fungal culture grew *Aspergillus fumigatus*. A CT scan of the chest revealed a crescent-shaped lucency (air crescent sign) with in the area of consolidation in the left upper lobe and right middle lobe [Figures 3 and 4]. Fiberoptic bronchoscopy showed purulent secretions coming from both upper lobe bronchi and left lower lobe bronchi. Bronchoalveolar lavage smears did not reveal any bacteria or acid-fast bacilli but the growth of *Aspergillus fumigatus*. Thus, he was diagnosed as a case of chronic necrotizing pulmonary aspergillosis with diabetes mellitus.

He was given insulin for glycemic control and oral itraconazole 200mg twice daily. After one month of itraconazole therapy, the patient started showing clinical improvement, became afebrile, gained weight, and the volume and the purulence of sputum also reduced considerably.

## DISCUSSION

Chronic necrotizing pulmonary aspergillosis (CNPA), also termed semi-invasive pulmonary aspergillosis (SIPA), is an uncommon, indolent pulmonary infection commonly seen in patients with altered local defense due to preexisting lung disease such as chronic obstructive pulmonary disease, bronchiectasis, pneumoconiosis and mildly compromised systemic defense due to diabetes mellitus, alcoholism, poor nutrition or low dose corticosteroid therapy.[1,2] Our patient was also a known case of diabetes mellitus.

This usually occur in middle-aged to older individuals who present with fever, productive cough, and weight loss that occurs over a period of months,[3] thereby mimicking tuberculosis and often resulting in a delay in the diagnosis (as happened in our case also). In contrast to the patients with simple mycetomas, CNPA nearly always presents with pulmonary or systemic symptoms. In addition, hemoptysis, the most common symptom in patients with mycetoma, is reported in only 10% of patients with CNPA and is rarely an isolated symptom.[3] Aspergilloma, a noninvasive form of aspergillosis, may develop in a healthy host, in which the organism colonizes a preexisting cavity whereas CNPA instead of developing in a preexisting cavity, it may create its own cavity in immunocompromised host and then grow as a relatively noninvasive organism. Radiographic manifestation usually consists initially of an area of consolidation in the upper lobe, which develops progressive cavitation over several weeks or months.[4] The cavitation may be associated with intracavitary soft tissue opacity which can be recognized radiographically with the appearance of an air crescent sign. The air crescent sign may be visualized on chest CT well before it is seen on the chest radiograph.

The diagnosis is suggested by the clinical course and the isolation of the fungus from pulmonary secretions; negative cultures for other pathogens and failure to respond to antibacterial or antimycobacterial therapy.[5] Diagnostic confirmation requires histologic evidence of local lung tissue invasion by septate hyphae, consistent with *Aspergillus* species; however, this is often difficult to obtain. Both transbronchial and percutaneous biopsy have low diagnostic yields for locally invasive aspergillosis when compared with autopsy findings.[1,5] Sputum is also unavailable for culture in many cases; even when present, the sensitivity of such culture is probably in the order of 50% to 60%.[6] Similar values have been found for culture of respiratory tract secretions sampled by bronchoalveolar lavage or bronchial washing or brushing. A positive sputum culture may also reflect simple colonization. However in the appropriate clinical setting, repeated positive sputum or BAL fluid cultures have been found to be a reliable method of diagnosis.[7]

Unlike *Aspergillus* fungal ball, in which medical therapy has very little proven benefit,[8] successful treatment of CNPA has been reported.[5] A treatment protocol utilizing itraconazole, followed, if necessary, by intravenous amphotericin B or surgical excision or intracavitatary amphotericin has been proposed.[2] The dose and duration of therapy should be based on clinical response.[3] Maintenance therapy with itraconazole can be considered in patients with residual parenchymal scarring.[3] Voriconazole and Micafungin are useful in refractory cases.[9] In this disorder, diagnosis is usually delayed, patients are poor operative candidates, and postoperative complications are common. Treatment outcome is likely to be influenced by the severity of co-morbid conditions, the extent of the underlying disease, delays in diagnosis, and initiation of effective therapy.[3]

In a country like India, cavity in upper zone of chest radiograph is considered a case of pulmonary tuberculosis even in the absence of AFB. Diagnosis of chronic necrotizing pulmonary aspergillosis must be considered in those patient in which disease progress along with evolution of fungus ball after a trial of antitubercular treatment.
